# Big MAC on the Interventional Menu

**DOI:** 10.1016/j.jscai.2025.103860

**Published:** 2025-07-22

**Authors:** Pedro Engel Gonzalez, Anna E. Bortnick

**Affiliations:** a Department of Medicine, Division of Cardiology, Henry Ford Health, Detroit, Michigan; b Department of Medicine, Division of Cardiology, Montefiore Medical Center and Albert Einstein College of Medicine, Bronx, New York

**Keywords:** calcification, mitral annular calcification, mitral valve

What happens to individuals with severe mitral annular calcification (MAC) over time and how many might be eligible for treatment is of interest as hospitals plan to implement or expand their mitral valve structural heart programs. There is no medical therapy for MAC, nor any well-established biomarkers of progression or cell or animal models of the disease.^1^ MAC is associated with increased risk of stroke, myocardial infarction, heart failure, and mortality.^2^^,^^3^ Surgical valve replacement or repair of the mitral valve in the presence of MAC is risky and challenging. Percutaneous treatment has been limited to off-label transcatheter aortic valve-in-MAC or trial devices, but recent US Food and Drug Administration (FDA) approval of the Tendyne transcatheter mitral valve replacement (TMVR) (Abbott) offers new promise to an undertreated patient population.

Like atherosclerosis, MAC arises from lipid-laden foam cells and coalescing calcific vesicles, stiffening tissue as it extends along the mitral valve leaflets until mitral stenosis (MS, 0.1%) or mitral regurgitation (MR, 1.7%) arises.^4^ MAC is highly prevalent among older individuals, especially women. MAC is reported in 8% to 15% of those aged >65 years, but >40% in some studies, depending on the cohort and method used to assess the mitral valve.^4^^,^^5^

In this issue of *JSCAI*, Mendez-Hirata et al^6^ describe the natural history of individuals with MAC with and without mitral valve dysfunction at a single center over 17 years and compare them with age-matched and sex-matched individuals without MAC, excluding those with heart transplantation or congenital heart disease. MAC was assessed by computed tomography, mostly done for individuals with severe aortic stenosis (AS) as part of preprocedural planning for transcatheter aortic valve replacement. Prime strengths of the study are that it evaluated a large real-world cohort and defined a cohort of individuals who might be eligible for treatment of MAC-associated mitral valve disease. The outcome of interest was all-cause mortality. The study highlights several key findings regarding MAC and its association with valvular dysfunction. First, it underscores the high prevalence of significant valvular disease in patients with MAC. Second, it delineates the poor clinical prognosis associated with this population, with dismal 2-year all-cause mortality rates of 26% and 21% in those with MR and MS, respectively. Third, it suggests that cardiovascular disease–related mortality may be attenuated in patients eligible for intervention: 26% in those with significant valvular disease who did not receive an intervention versus 19% in those who did. An intervention for the mitral valve was performed in 17.2% of those with greater than or equal to moderate mitral valve disease. The reason for undertreatment may have been due to individuals being poor candidates for transcatheter edge-to-edge repair or surgery or declining invasive options, and the study was not able to assess these factors. Indeed, those with MAC had a higher burden of comorbidities than those without.

While mitral interventions evolved over the study period, individuals with severe MAC would have been excluded from transcatheter edge-to-edge repair for severe MR, valve-in-MAC was not common, and trials for dedicated TMVR devices were not widespread. The study evaluated a single center in the Midwest and could not account for individuals who may have migrated out from the site. Other competing risks or comorbidities may not have made it possible for those individuals with severe MAC to be treated, and the majority were being evaluated for concomitant AS. Additional, prospective data are needed to better define and track the population with MAC that structural heart programs should be targeting for TMVR with dedicated devices.

The findings must be interpreted with caution. The observational nature of the study, the heterogeneity of interventions performed, and the small proportion of patients who underwent procedures limit the ability to draw causal inferences regarding mortality reduction; however, this signal raises a promising hypothesis that should be explored further—specifically, whether timely intervention in select patients with MAC can truly improve outcomes.

On the horizon, new technologies are generating optimism. The Tendyne TMVR system has received FDA approval for patients with MAC based on the SUMMIT trial (NCT04940390), which includes a MAC cohort. The device also holds CE Mark approval (2020). Tendyne is a trileaflet porcine bioprosthetic valve housed in dual frames, delivered via an apical approach, and anchored by a tether secured with an apical pad. This design allows for repositioning and retrieval during the procedure—an advantage in anatomically complex patients. Although the 1-year outcomes for the MAC cohort are not yet available, baseline and 30-day data are encouraging.^7^ At baseline, 89% had severe MR (grade III or IV), and 10.7% had severe MS. Technical and procedural success rates were 94% and 98%, respectively. At 30 days, 98.9% had trace or no MR, 68.6% were New York Heart Association class I or II, and mortality was 6.8%; however, complications were nontrivial: 21.4% experienced major bleeding, 2.9% required cardiopulmonary bypass, and 2% needed mitral reintervention. Additionally, the screen failure rate was high (75%), largely due to anatomical exclusion criteria such as insufficient predicted neo-left ventricular outflow tract (neo-LVOT), annular dimensions outside the acceptable range, or interference with the valve’s inner frame.

The Achilles heel of TMVR in MAC remains the challenge of a small predicted neo-LVOT, which precludes many patients from receiving transcatheter therapies. The SUMMIT trial did not permit anterior leaflet modification, which could potentially improve eligibility. Techniques like intentional laceration of the anterior mitral leaflet have shown safety and efficacy at expert centers,^8^ and newer, simplified methods are under investigation like balloon-assisted translocation of the mitral anterior leaflet. Pre-emptive alcohol septal ablation has also been demonstrated to be a safe and predictable means of increasing neo-LVOT and potentially expanding candidacy.^9^

Clearly, there is an urgent need to broaden the therapeutic armamentarium for patients with MAC and with significant valve disease. Valve-in-MAC—TMVR with a SAPIEN 3 transcatheter heart valve (Edwards Lifesciences)—remains off-label and is not currently approved by FDA, yet utilization is increasing despite results from the Mitral Implantation of TRAnscatheter vaLves (MITRAL) trial, which showed high 1-year mortality in valve-in-MAC patients.^10^ Results from the ongoing MITRAL II trial (NCT04408430) are eagerly awaited and may demonstrate improved outcomes with a transseptal approach and modern LVOT protection strategies such as leaflet laceration or septal ablation.

Other TMVR platforms also show promise. The ENCIRCLE trial (NCT04153292) is evaluating the SAPIEN M3 (Edwards Lifesciences), which uses a nitinol docking system to encircle the mitral leaflets and anchor the valve, incorporating a polyethylene terephthalate skirt to minimize paravalvular leak. Meanwhile, the AltaValve (4C Medical) uses a self-expanding nitinol stent frame anchored in the left atrium that houses a 27.0-mm trileaflet bovine pericardial valve. Its unique design avoids rigid anchoring to the native mitral apparatus and minimizes ventricular protrusion, which may benefit patients at risk of LVOT obstruction. The early feasibility study showed promising results,^11^ and the pivotal trial is currently underway.

While the aortic valve has been the focus of the last decade, the mitral valve may be the focus of the next. The field of TMVR for MAC is evolving rapidly, and as Mendez-Hirata et al^6^ showed, there is a population of undertreated patients. While significant anatomical and procedural challenges remain, innovation in device design and improvement in procedural techniques is steadily expanding the treatment landscape for this high-risk population.
